# Proximal Humerus Fractures: Reliability of Neer Versus AO Classification on Plain Radiographs and Computed Tomography

**DOI:** 10.7759/cureus.8520

**Published:** 2020-06-09

**Authors:** Michael Stoddart, Oliver Pearce, James Smith, Philip McCann, Barnaby Sheridan, Khalid Al-Hourani

**Affiliations:** 1 Trauma and Orthopaedics, Royal National Orthopaedic Hospital, London, GBR; 2 Trauma and Orthopaedics, Southmead Hospital, Bristol, GBR; 3 Orthopaedics, Southmead Hospital, Bristol, GBR; 4 Orthopaedics, Musgrove Park Hospital, Taunton, GBR; 5 Orthopaedics, The Royal Infirmary of Edinburgh, Edinburgh, GBR

**Keywords:** proximal humerus fracture, classification, interobserver reliability, neer, ao

## Abstract

Introduction: Several classifications for proximal humeral fractures exist, with excellent reliability and reproducibility of such classifications being a desirable feature. Despite their widespread use, these systems are variable in both reliability and accuracy. We aimed to, a) assess and compare the reliability of the Neer (complete and abbreviated versions) and Arbeitsgemeinschaft für Osteosynthesefragenbeing (AO) classifications, and b) identify if computed tomography (CT) made any difference to the reliability of Neer and AO classifications when compared to plain radiographs alone.

Materials and methods: This is a single-centre retrospective study identifying all proximal humeral fractures presenting between February 2016 and February 2017 as a result of trauma that subsequently proceeded to CT. Two specialty orthopaedic trainees analysed the plain radiographs as well as CT images over two rounds, spaced two weeks apart. The Neer 16-grade, abbreviated Neer 6-grade and AO classifications were used. Intra- and inter-observer reliability of each classification system was assessed using the Kappa coefficient.

Results: Twenty-two patients were included. The mean age was 62 years (SD 14.5). Management changed in 9/22 patients based on CT. Computed tomography changed Neer-16 type in 16% observations, Neer-6 in 10%, and AO in 23%. This was significant when comparing Neer-6 and AO classifications (p = 0.04). Neer-6 had the best inter-observer reliability (0.737) with the management of one patient changing after CT. On X-ray and CT, intra-observer agreement was substantial, >0.7, using Neer-16 and Neer-6 (p<0.005). Inter-observer agreement for Neer-16 and Neer-6 was substantial, >0.7 (p<0.005). In comparison, intra- and inter-observer agreements for AO were lower on X-ray and CT, 0.4-0.6, (p<0.005).

Conclusion: Our study shows that simplicity is key with a high degree of reliability in the abbreviated Neer classification. Computed tomography allowed greater reliability than radiographs in classifying fractures, affecting management decisions in 41% of patients. The comprehensive Neer classification showed similar intra- and inter-observer reliabilities to AO.

## Introduction

A standardised classification system serves multiple purposes when applied to fractures. They aid communication between medical professions and allow standardisation in research. Perhaps more importantly they can be used in prognostication and to guide management and intervention when necessary [1].

Proximal humeral fractures account for 6% of all fractures in the Western World and are the third most common osteoporotic fracture [2,3]. As the majority occur in patients older than 65, they represent a significant burden of disease within the aging UK population [4]. 

A number of classification systems of the proximal humerus are described in the literature, with the Neer and Arbeitsgemeinschaft für Osteosynthesefragenbeing (AO) the most widely used [5,6]. A simpler modified Neer system using only six fracture types is described by Bernstein but is not in common use [7]. The criteria for displacement in this system remains as per Neer’s original classification (more than one centimetre of displacement or 45 degrees of angulation).

Despite the widespread use of these classifications, they have been shown to be variable with regards to both their reliability and accuracy [8-10]. Following the introduction of cross-sectional imaging, there have been attempts at validating these classification systems. Both Bernstein and Sjoden reported no improvement with two or three-dimensional computed tomography (CT) imaging, however, Brunner found that the use of more sophisticated three-dimensional modelling improved both inter and intra-observer reliability [7,11-13]. 

We aimed to, a) assess and compare the reliability of the Neer (complete and Neer-6 versions) and AO classifications, and b) identify if CT made any difference to the reliability of Neer and AO classifications when compared to plain radiographs alone.

## Materials and methods

This is a single centre retrospective cohort study conducted between Feb 2016 and Feb 2017 on all patients presenting with proximal humeral fractures to a district general hospital. Those patients who underwent X-ray and subsequent CT were included in the study. Patient demographics (sex, age) were collected from clinical notes. The mechanism of injury was divided into high and low energy, and direction of dislocation (where appropriate) were also noted. Patients with pathological fractures were excluded.

Two specialty orthopaedic trainees acted as observers. The presenting shoulder trauma series, consisting of anteroposterior, lateral and axillary views, were classified according to the Neer 16-grade, AO, and a modified Neer 6-grade classification system. This modified Neer 6-grade, as described by Bernstein, included six types of fractures one, two, three and four-part fractures, fracture dislocations, and articular fractures (Table 1). The CT images were then scrutinised by the two observers, and the fracture classified using the same systems. Discrepancies between the classification using plain radiographs and CT images were noted. This process was repeated by each trainee two weeks later with blinding of the first round of results. Four rounds of classification were therefore undertaken. In cases of disagreement of classification type between the two reviewers, the fracture pattern was classified following mutual agreement with involvement of a senior author for consensus. Statistical accuracy testing was not performed. 

**Table 1:** Breakdown of Neer classification

Fracture type	Neer 16 classification type	Modified Neer 6 classification type
1 part fracture	1	1
2 part fracture	2, 3, 4, 5	2
3 part fracture	8, 9	3
4 part fracture	12	4
Fracture dislocation	6, 7, 10, 11, 13, 14	5
Articular surface fracture	15, 16	6

The management decision for each injury was based on the initial presenting plain radiograph and associated fracture classification. However, if the classification and therefore management plan changed following cross-sectional imaging, this was noted. Reliability was therefore assessed against CT being the gold standard. 

Analysis was undertaken with IBM “SPSS” statistics version 23 (IBM Corp, Armonk, NY). Intra- and inter-observer reliability was assessed using the Kappa coefficient. Interpretation of agreement uses the Landis and Koch reference values, where a Kappa value of < 0 indicates no agreement, 0-0.20 as slight agreement, 0.21-0.40 as fair agreement, 0.41-0.60 as moderate agreement, 0.61-0.80 as substantial agreement and 0.81-1 as almost perfect observer agreement [14].

Chi-squared test was used to evaluate statistical significance between groups. We assumed a-priori that a p value of less than 0.05 was significant.

## Results

Twenty-three patients were identified with one excluded as a result of a pathological fracture. A total of twenty-two patients were therefore eligible for final analysis. Mean age was 62 years (SD 14.5), with 5 males and 17 females, all were closed injuries. Six were dislocated on their presenting plain radiographs. Table 2 demonstrates patient demographics and injury details.

**Table 2:** Patient demographics

Demographic	
Sex	5 Male / 17 Female
Age (Standard Deviation)	62 (14.5)
Side	10 Right / 12 Left
Energy	19 Low / 3 High
Dislocated	6
Operated	6

The Neer-16, Neer-6 demonstrated good agreement for intra-observer reliability, ranging from 0.668 to 0.740 on plain X-ray and from 0.57 to 0.79 on CT. In comparison, intra-observer agreements for AO were lower on X-ray and CT, 0.4-0.6 (Table 3). 

**Table 3:** Kappa coefficients for intra-observer reliability

	Neer-16		Neer-6		AO	
	Observer 1	Observer 2	Observer 1	Observer 2	Observer 1	Observer 2
X-ray	0.68	0.73	0.67	0.74	0.41	0.60
CT	0.57	0.79	0.68	0.76	0.42	0.60

The Neer-6 classification demonstrated the greatest inter-observer reliability (0.74) on plain film, with only moderate agreement (0.56) when using AO. The inter-observer agreement showed similar reliability when using CT images for all three classification systems (Table 4). 

**Table 4:** Kappa coefficients for inter-observer reliability

	Neer-16	Neer-6	AO
X-ray	0.702	0.737	0.557
CT	0.705	0.690	0.589

Following review of cross sectional imaging, 15.9% (14/88) of the observations changed when using the Neer-16 type classification. 10.2% (9/88) changed using the Neer-6 type and 22.7% (20/88) changed if the AO classification system was used. This change in classification was significant when comparing Neer-6 and AO systems (p < 0.05). Overall, management changed in 9/22 patients following CT as a result of improved delineation of fracture pattern. In particular, only one (1/22, 4.5%) patient’s management changed when comparing Neer-6 on plain radiograph and CT (Table 5). This was significant (p<0.05). 

**Table 5:** Change in classification and management following CT

	Number	Percentage	Change in Management
Neer-16	14/88	16%	3
Neer-6	9/88	10%	1
AO	20/88	23%	8

## Discussion

Our results show that when classifying proximal humeral fractures from plain X-rays there is greater intra- and inter-observer agreement if the Neer-6 and Neer-16 systems are used compared to the AO system. This was also true when classifying proximal humeral fractures using cross-sectional imaging.

With regards to both the intra- and inter-observer reliability, there was substantial agreement when using the Neer-16 and Neer-6 classifications, and moderate agreement with the AO classification. Our results demonstrated greater levels of agreement than other articles in the literature. In particular, Bernstein et al. (k = 0.52), Siebenrock et al. (k = 0.40), and Sidor et al. (k = 0.48) found levels of fair to moderate agreement when classifying proximal humeral fractures using the methods described [7-9]. 

One of the reasons posed for the lower agreement is due to the high number of categories in the retrospective classification groups [15,16]. Bernstein reported that the modified Neer-6 classification sacrificed information by using only 6 types of fractures (compared to 16) with no improvement in reproducibility [7]. This was also found by Sidor with no improvement to either inter or intra-observer kappa values [9]. In contrast, we found that the modified Neer-6 classification demonstrated substantial agreement between observers and remained accurate following CT. This was found to be statistically significant compared to AO classification, with over 20% of observed classifications changing on reviewing cross-sectional imaging. Additionally, if plain radiography was the only imaging modality to be used, Neer-6 was most accurate in dictating a definitive management plan for the patients studied, with only one patient’s management changing based on CT. This would be particularly relevant in healthcare systems where there is limited access to cross-sectional imaging. 

This study is limited by its relatively small sample size. However, as it is a pragmatic observational study, this represents the normal case load in our department. Despite small numbers, analysis demonstrated statistical significance with excellent levels of agreement. It has been shown that the reliability improves with more experienced observers and that training improves the reliability of fracture classification [9,10]. However, we believe the variable level of experience and seniority of our observers gives more generalizable results and can more readily be applied to everyday practice. The Neer classification is the standard classification in our department, which may have led to a bias in the results. It is important to note that our study is an evaluation of the reliability of the classifications described and not an accuracy study. As previously described, the decision for operative versus non-operative management is multifactorial, a fact that could make our findings less valid. 

Our observers did not have access to real 3D imaging and modelling described by Brunner, who demonstrated a consistent increase in inter-observer agreement, challenging those who concluded that CT scanning adds little to assessment and classification [13]. By including only those patients who had a subsequent CT in our study, this may have led to a bias of more complex fracture patterns, and so more difficult to classify solely using plain radiographs.

The utility of a fracture classification system is determined by its ability to predict clinical results and to guide prognosis. Some studies have attempted to correlate between both Neer and AO classifications to functional outcome scores thus providing the treating surgeon with information to direct management and further investigation [17,18]. However, this is particularly difficult in proximal humeral fractures as there are multiple factors beyond the fracture pattern that influence outcomes, including age, comorbidities, bone stock, and reduction quality [16,19]. Newer systems, such as Codman’s and Resch’s classifications, have been shown to have higher reliability and prognostic value for the indication and outcomes of proximal humeral fractures [20-22]. Despite this, the Neer and AO classifications remain the most commonly used classification systems. There is increasing level 1 evidence that more complex fractures can be managed conservatively, with no significant difference in outcome scores [23,24]. With this in mind, it is therefore important to have a robust classification system in order to accurately stage and prognosticate these injuries.

## Conclusions

Accurate and consistent classification of proximal humeral fractures remains difficult. The Neer and AO systems remain the most widely used classification systems despite their variability and moderate reliability. Our study demonstrated significantly higher reliabilities using Neer classifications compared to AO. The abbreviated Neer-6 classification, which requires a simple modification of a well-known system, is significantly more clinically accurate and reliable than AO. The authors recommend using the abbreviated Neer-6 classification for reliability and reproducibility in proximal humerus fractures.
